# Endometrial biopsy under direct hysteroscopic visualisation versus blind endometrial sampling for the diagnosis of endometrial hyperplasia and cancer: Systematic review and meta-analysis

**DOI:** 10.52054/FVVO.14.2.023

**Published:** 2022-07-01

**Authors:** A Di Spiezio Sardo, G. Saccone, J Carugno, L.A. Pacheco, B Zizolfi, S Haimovich, T.J. Clark

**Affiliations:** Department of Public Health, School of Medicine, University of Naples Federico II, Naples, Italy; Department of Neuroscience, Reproductive Science, and Dentistry, School of Medicine, University of Naples Federico II, Naples, Italy; Obstetrics, Gynecology and Reproductive ScienceDepartment, Minimally Invasive Gynecology Division, University of Miami, Miami, FL, USA; Unidad de Endoscopia Ginecológica, Centro Gutenberg, Málaga, Spain; Hillel Yaffe Medical Center, Technion-Israel Technology Institute, Hadera, Israel; School of Clinical and Experimental Medicine, College of Medical and Dental Sciences, University of Birmingham, Birmingham, UK

**Keywords:** endometrial cancer, endometrial hyperplasia, endometrial biopsy, hysteroscopy, diagnosis

## Abstract

**Background:**

Endometrial cancer is the most common gynaecological neoplasia in western countries. Diagnosis of endometrial cancer requires an endometrial biopsy. A good quality endometrial biopsy allows not only the identification of the pathology, but also preoperative histologic subtype classification. Endometrial biopsy can be performed under direct hysteroscopic visualisation, but also using blind sampling techniques

**Objectives:**

To compare endometrial biopsy performed under direct hysteroscopic visualisation versus blind sampling for the diagnosis of endometrial hyperplasia and cancer.

**Materials and methods:**

Systematic review and meta-analysis. Electronic databases were searched from their inception until March 2022.We included all studies comparing endometrial biopsy performed under direct hysteroscopic visualisation versus blind endometrial sampling.

**Main outcome measures:**

Sample adequacy, failure rate to detect endometrial cancer or endometrial hyperplasia, and rate of detection of endometrial cancer. The summary measures were reported as relative risk (RR) with 95% of confidence interval (CI).

**Results:**

Four studies with a total of 1,295 patients were included. Endometrial biopsy under direct hysteroscopic visualisation was associated with a significantly higher rate of sample adequacy (RR 1.13, 95% CI 1.10 to 1.17), and significantly lower risk of failure to detect endometrial cancer or endometrial hyperplasia (RR 0.16, 95% CI 0.03 to 0.92) compared to blind endometrial sampling. However, there was no significant difference between endometrial biopsies taken under direct hysteroscopic visualisation or blindly, with or without a preceding diagnostic hysteroscopy, in the rate of detection of endometrial cancer (RR 0.18, 95% CI 0.03 to 1.06).

**Conclusion:**

Hysteroscopic endometrial biopsy under direct visualisation is associated with significantly higher rate of sample adequacy and is comparable to blind endometrial sampling for the diagnosis of endometrial cancer and precancer.

**What is new?:**

Hysteroscopic endometrial biopsy under direct visualisation would be expected to reduce diagnostic failure for endometrial cancer compared to blind endometrial sampling.

## Introduction

Endometrial cancer is the most common gynaecological neoplasia in western countries (Randall, 2019). Worldwide, every year more than 350,000 new cases are diagnosed (Ferlay et al., 2019). Endometrial cancer is often diagnosed at an early stage because it frequently causes abnormal vaginal bleeding that prompts timely clinical evaluation (Lu and Broaddus, 2020).

The evaluation of women at risk for endometrial cancer includes transvaginal ultrasound (Jónsdóttir et al., 2021), but the diagnosis requires endometrial biopsy. A good quality endometrial biopsy allows not only the identification of the pathology, but also preoperative histologic subtype classification (Da Cruz Paula et al., 2021). Endometrial biopsy can be performed under direct hysteroscopic visualisation, but also using blind sampling techniques (Di Spiezio Sardo et al., 2020; Papalona et al., 2015; Narice et al., 2018; Rauf et al., 2014). It is unclear whether hysteroscopic biopsy or blind endometrial sampling is superior in detecting significant endometrial disease, endometrial cancer, and endometrial hyperplasia with or without atypia. We therefore conducted a systematic review of the literature to investigate the diagnostic performance of endometrial biopsy performed under direct hysteroscopic visualisation versus blind sampling for diagnosis of endometrial pathology.

## Methods

### Search strategy and selection criteria

This systematic review and meta-analysis were conducted according to a protocol designed a priori and recommended for systematic review (Slim et al., 2003). The meta-analysis was reported following the Preferred Reporting Item for Systematic Reviews and Meta-analyses (PRISMA) statement (Moher et al., 2009). Before data extraction, the review was registered into the PROSPERO International Prospective Register of Systematic Reviews (registration No.: CRD42021245668).

The following electronic databases MEDLINE, Scopus, ClinicalTrials.gov, EMBASE, ScienceDirect, the Cochrane Library at the CENTRAL Register of Controlled Trials, and Scielo were searched from their inception until March 2022. Search terms used were “endometrial cancer”, “hysteroscopy”, and “biopsy”. No restrictions for language or geographical location were applied. In addition, the reference lists of all identified articles were examined to identify studies not captured by electronic searches. The electronic search and the eligibility of the studies were independently assessed by two authors (GS, ADS). Differences were discussed until a consensus was reached.

We included all studies comparing endometrial biopsy performed under direct hysteroscopic visualisation versus blind endometrial sampling for the diagnosis of endometrial cancer or pre-cancerous endometrial pathologies (endometrial hyperplasia with or without atypia). Both observational and randomised trials were included in the review. We planned to include all hysteroscopic settings and all hysteroscopic techniques, e.g., grasp technique, mechanical tissue removal systems or monopolar/ bipolar energy resection. Studies comparing different hysteroscopic techniques but with no blind sampling as a control group, were excluded. The control group included all types of endometrial sampling methods, such as the use of miniature biopsy devices (e.g., Pipelle®, suction biopsy, Novak curette, vacuum aspiration) and blind dilation and curettage (D&C). We also included studies that used hysteroscopic oriented biopsy in the blind sampling group. Hysteroscopic oriented biopsy was defined as a biopsy performed using a blind technique immediately after a diagnostic hysteroscopy. Studies comparing different blind techniques, e.g., Pipelle® vs D&C, with no hysteroscopic approach as intervention group were excluded. Case reports and studies including less than 5 patients were excluded.

### Data extraction and risk of bias assessment

Two reviewers (ADS, GS) independently assessed the risk of bias of the included studies via the Methodological Index for Non-Randomized Studies (MINORS) (Slim et al., 2003). Seven domains related to risk of bias were assessed in each study: 1) Aim (clearly stated aim), 2) Rate (inclusion of consecutive patients and response rate), 3) Data (prospective collection of data), 4) Bias (unbiased assessment of study endpoints), 5) Time (follow-up time appropriate), 6) Loss (loss to follow-up), 7) Size (calculation of the sample size). Review authors’ judgments were categorised as “low risk,” “high risk” or “unclear risk of bias.” Discrepancies were resolved by discussion with a third reviewer (BZ). Additional data were asked from the authors of the original studies, if feasible.

### Primary and secondary outcomes

All analyses were done using an intention-to-treat approach, evaluating women according to the treatment group to which they were randomly allocated in the original study. The primary outcome was sample adequacy, defined as enough tissue quantity and quality to be analysed by pathologists. The secondary outcomes were failure to detect endometrial cancer or endometrial hyperplasia (McCluggage, 2006), and mean procedure length for sampling.

### Statistical analysis

The data analyses were completed using Review Manager v. 5.3 (The Nordic Cochrane Centre, Cochrane Collaboration, 2014, Copenhagen, Denmark). The summary measures were reported as summary relative risk (RR) or as summary mean difference with 95% of confidence interval (CI) using the fixed effects model. I-squared (Higgins I2) greater than 0% was used to identify heterogeneity. Data from each eligible study were extracted without modification of original data onto custom-made data collection forms. 2 by 2 contingency tables were constructed and relative risks (RR) calculated. For continuous outcomes means ± standard deviation (SD) was extracted and imported into Review Manager. Potential publication biases were assessed statistically by using Begg and Egger’s tests. A p value <0.05 was considered

## Results

### Study selection and study characteristics

**Figure 1:**
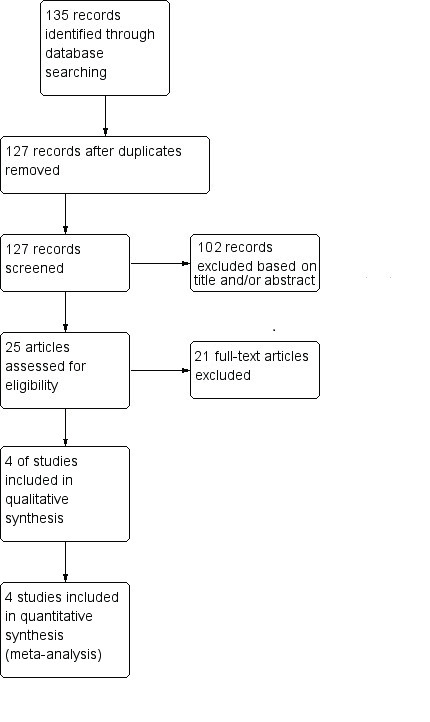
Flow diagram of studies identified in the systematic review. (Prisma template [Preferred Reporting Item for Systematic Reviews and Meta-analyses]).

The flow of study identification is shown in Figure 1. A total of 25 studies were identified as relevant and screened (Supplementary Table I) (Spiezio Sardo et al., 2020; Goldberg et al., 1982; Batool et al., 1994; Ben-Baruch et al., 1994; Van de Bosch et al., 1995; Van de Bosch et al., 1996; Giusa-Chiferi et al., 1996; Gupta et al., 1996; De Silva et al., 1997; Mortakis and Mavrelos, 1997; Bunyavejchevin et al., 2001; Epstein et al., 2001; Spicer et al., 2006; Rauf et al., 2004; Liu et al., 2015; Critchley et al., 2004; Henig et al., 1989; Polena et al., 2007; Tahir et al., 1999; Cooper and Erickson, 2000; Rosenblatt et al., 2017; Yela et al., 2018; Wanderley et al., 2016; Li et al., 2017; Ceci et al., 2002). Of those, 21 studies were excluded: 12 because they used blind procedures both in the intervention and in the control group without hysteroscopy (Goldberg et al., 1982; Batool et al., 1994; Ben-Baruch et al., 1994; Van de Bosch et al., 1995; Van de Bosch et al., 1996; Gupta et al., 1996; Bunyavejchevin et al., 2001; Epstein et al., 2001; Rauf et al., 2004; Liu et al., 2015; Critchley et al., 2004; Henig et al., 1989); two studies were excluded because women underwent endometrial biopsy under direct hysteroscopic visualisation in both intervention and control group (Giusa-Chiferi et al., 1996; Tahir et al., 1999); four studies were excluded because patients underwent blind endometrial biopsy with Pipelle® followed by hysteroscopy (De Silva et al., 1997; Mortakis and Mavrelos, 1997; Spicer et al., 2006; Polena et al., 2007); Cooper and Erickson (2000) was excluded because it was a review; the study by Yela Da et al. (2018) was excluded because it compared patients undergoing endometrial biopsy under direct hysteroscopic visualisation with patients having transvaginal ultrasound; Li et al. (2017) was excluded because they used SAP-1 sampler device followed by hysteroscopy or D&C.

**Table I:** Characteristics of the studies assessed for eligibility.

	Study design	Index test	References standard	Study assessment
Goldberg 1982	Prospective cohort study	Vabra & Accurette	Blind D&C	Excluded
Henig 1989	RCT	Pipelle	Novak	Excluded
Batool 1994	Prospective cohort study	Pipelle	Blind D&C	Excluded
Ben-baruch 1994	Retrospective cohort study	Pipelle	Blind D&C	Excluded
Van den Bosch 1995	Prospective cohort study	Pipelle	Hysteroscopy w/histology	Excluded
Van den Bosch 1996	Prospective cohort study	Pipelle	Hysteroscopy w/histology	Excluded
Giusa-Chifieri 1996	Prospective cohort study	Novak	Hysteroscopy w/histology	Excluded
Gupta 1996	Prospective cohort study	Pipelle	Hysteroscopy w/histology	Excluded
De Silva 1997	Prospective cohort study	Pipelle	Hysteroscopy w/histology	Excluded
Mortakis 1997	Not reported	Pipelle	Hysteroscopy w/histology	Excluded
Tahir 1999	RCT	Inpatient hysteroscopy & D&C	Outpatient Pipelle ± TVU ± hysteroscopy	Excluded
Cooper 2000	Review	Directed biopsy with hysteroscopy	-	Excluded
Bunyavejchevin 2001	Prospective cohort study	Pipelle	Blind D&C	Excluded
Epstein 2001	Prospective cohort study	Endorette	Blind D&C	Excluded
Ceci 2002	Retrospective cohort study	Hysteroscopy	D&C*	Included
Critchley 2004	RCT	Pipelle	Tao Brush	Excluded
Spicer 2006	Prospective cohort study	Accurette	Hysteroscopy w/histology	Excluded
Polena 2007	Prospective sequential cohort study	Pipelle Mark 2	Pipelle Mark 2 ± hysteroscopy	Excluded
Rauf 2014	RCT	Pipelle	D&C	Excluded
Liu 2015	Prospective sequential cohort study	Pipelle	D&C	Excluded
Wanderley 2016	Cross-sectional study	Hysteroscopy	D&C	Included
Rosenblatt 2017	Prospective pilot study	MyoSure Lite hysteroscopic tissue removal system	D&C	Included
Li 2017	Prospective cohort study	SAP-1 sampler device followed by hysteroscopy (169)	SAP-1 sampler device followed by D&C (13)	Excluded
Yela 2018	Retrospective cohort study	TVU	Hysteroscopy	Excluded
Di Spiezio Sardo 2020	Retrospective cohort study	D&C	Hysteroscopy	Included

D&C, dilation, and curettage; RCT, randomised clinical trial; TVU, transvaginal ultrasound; *Control group was from Bettocchi et al. 2001 (37).

**Figure 2:**
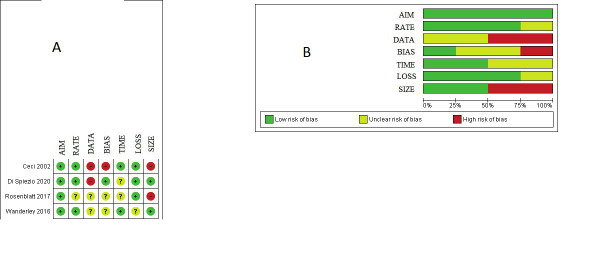
Assessment of risk of bias. Aim, clearly stated aim; Rate, inclusion of consecutive patients and response rate; Data, prospective collection of data; Bias, unbiased assessment of study end points; Time, follow-up time appropriate; Loss, loss to follow-up; Size, calculation of the sample size. (A) Summary of risk of bias for each study. Plus sign, low risk of bias; minus sign, high risk of bias; question mark, unclear risk of bias. (B) Risk of bias graph about each risk of bias item presented as percentages across all included studies.

Therefore, 4 studies (Ceci et al., 2002; Wanderley et al., 2016; Rosenblatt et al., 2017; Di Spiezio Sardo et al., 2020) with a total of 1,295 participants, were included in the meta-analysis. Publication bias was assessed statistically by using Begg’s and Egger’s tests, showed no significant bias (P=0.69 and P=0.51, respectively). The quality of the studies included in our meta-analysis is reported in Figure 2. All the included studies were judged as low risk of bias in ‘aim’ but the risk of bias for all other domains was high or unclear. Authors of two studies (Ceci et al., 2002; Di Spiezio Sardo et al., 2020) were contacted where data were missing, or unclear and additional unpublished data were obtained.

Table I shows the characteristics of the included studies. All studies used hysterectomy as the diagnostic reference standard except for Wanderley et al. (2016) where the reference standard was not reported. The indications for hysterectomy were suspected cancer in one study (Di Spiezio Sardo et al., 2020); abnormal bleeding, polyps or a postmenopausal endometrial thickness (ET) >4mm in one study (Ceci et al., 2002); abnormal bleeding, or a postmenopausal ET >4mm or premenopausal ET >15mm in one study (Wanderley et al., 2016); while the indication was not reported in one study (Rosenblatt et al., 2017). All studies included women of pre- and postmenopausal status, apart from Rosenblatt et al. (2017) which restricted recruitment to postmenopausal women only.

It should be noted that the retrospective study by Ceci et al. (2002) included 443 patients who underwent office hysteroscopy followed by hysterectomy. The results of this study were then compared with a historical control of a previous study in which the same group of researchers examined the diagnostic accuracy of dilatation and curettage (D&C) with hysterectomy as the diagnostic reference standard (Bettocchi et al., 2001).

### Synthesis of results

**Figure 3:**
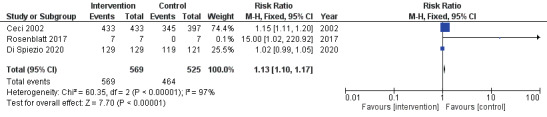
Forest plot for sample adequacy.

**Figure 4:**
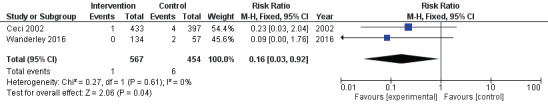
Forest plot for the risk of failure to detect endometrial cancer or endometrial hyperplasia.

**Figure 5:**
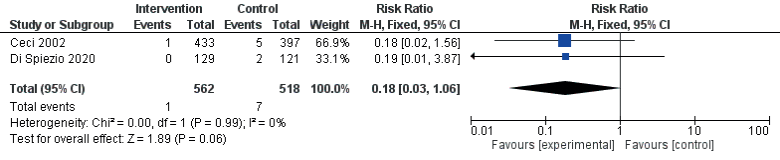
Forest plot for the risk of failure to detect endometrial cancer.

Figure 3 and Figure 4 show the forest plots for primary and secondary outcomes. Endometrial biopsy under direct hysteroscopic visualisation was associated with significantly higher rate of sample adequacy (RR 1.13, 95% CI 1.10 to 1.17; Figure 3), although there was considerable statistical heterogeneity (I2=97%). There was a significantly lower risk of failure to detect endometrial cancer or endometrial hyperplasia (RR 0.16, 95% CI 0.03 to 0.92; I2=0%; Figure 4) compared to blind sampling. There was no significant difference between endometrial biopsies taken under direct hysteroscopic visualisation or blindly, with or without a preceding diagnostic hysteroscopy, in the rate of detection of endometrial cancer (RR 0.18, 95% CI 0.03 to 1.06: Figure 5). Whilst the point estimate for detection of endometrial cancer favoured direct hysteroscopic biopsy, the data were derived from two studies only and statistical significance was not reached (Figure 5). No differences were found in the mean procedure length for sampling (44± vs 47±38 seconds; mean difference -3.00 seconds, 95% CI -35.91 to 29.91).

## Discussion

### Main findings

This systematic review aimed to compare sample adequacy and failure rates of endometrial biopsy performed under direct hysteroscopic visualisation versus blind endometrial sampling for the diagnosis of endometrial hyperplasia and cancer. Four studies (Ceci et al., 2002; Wanderley et al., 2016; Rosenblatt et al., 2017; Di Spiezio Sardo et al., 2020), with a total of 1,295 participants, were included in the meta-analysis. Endometrial biopsy under direct hysteroscopic visualisation was associated with significantly higher rate of sample adequacy compared to blind sampling.

Hysteroscopic visualisation was also associated with 82% decreased risk of failure to detect endometrial cancer, although statistical significance was not reached (p=0.06). Pooled data did not report any significant differences in the mean procedure length for sampling between the two techniques, with a mean of about 44-47 seconds.

### Strengths and Limitations

We conducted a comprehensive search and followed standard approaches to conducting a systematic quantitative review (Cumpston et al., 2019).

However, findings from this systematic review and meta-analysis are limited by the observational non- randomised study design of the studies included. Of the four studies that were included in the final analysis only one had a prospective study design (Rosenblatt et al., 2017). The source studies were heterogeneous, limiting the ability to draw meaningful conclusions from the pooled analyses. The main limitation of the review was the low quality of the included studies. In particular, one of the included (Wanderley et al., 2016) studies did not report the reference standard used to evaluate the methods of endometrial sampling against. Considering the methodological deficiencies of the primary studies we were unable to construct 2x2 contingency tables to assess overall diagnostic accuracy.

### Implication

Endometrial carcinoma is the most common gynaecological cancer in western countries. After history taking and physical examination, the first step in the workup of a patient with suspected endometrial cancer is usually transvaginal ultrasound, followed by endometrial biopsy. A good quality endometrial biopsy allows not only the diagnosis of endometrial cancer but also the histologic subtype classification. Currently, there is a variety of endometrial sampling methods, including blind sampling with Pipelle®, blind D&C, hysteroscopy-oriented biopsy, or hysteroscopic endometrial biopsy under direct visualisation. Diagnostic accuracy studies of endometrial cancer showed high diagnostic accuracy when the endometrial biopsy is obtained under direct hysteroscopic visualisation (Clark et al., 2002), and low to moderate when collected by blind D&C (Bettocchi et al., 2001; Vorgias et al., 2003). A large number of papers have extensively shown throughout the years the significant limitations of the blind technique, including the need for in-patient admission and general or regional anaesthesia; the high risk of complications; poor diagnostic accuracy (high number of focal lesions missed); and the total absence of any therapeutic role (Bettocchi et al., 2001).

However, despite this evidence, the Society of Gynecologic Oncology and the American Congress of Obstetricians and Gynecologists still emphasise the diagnostic and therapeutic role of D&C (Practice Bulletin No 149, 2015). Notably, when dealing with endometrial cancer, it is important to distinguish between diffuse or focal cancer (Patel et al., 2010). Indeed, the value of any blind procedure is when it reports a positive result, when it is negative (especially in cases of focal pathology or early adenocarcinoma) it can be a false negative and therefore hysteroscopy may be required (Clark, 2017; van Hanegem, 2017).

It is possible that failure to adopt hysteroscopically directed endometrial biopsy reflect the need to take multiple samples requiring several instrument insertions due to the small amount of tissue obtained with conventional 5Fr / 7Fr forceps. However, with the introduction of mechanical hysteroscopic tissue removal (mHTR) systems, large, representative endometrial tissue samples can easily be obtained without the need for repeated reinsertion of the hysteroscope (Franchini 2021). Robust, diagnostic accuracy studies are needed to compare the accuracy of mHTR against blind endometrial sampling and / or conventional hysteroscopic sampling methods.

## Conclusion

In summary, hysteroscopic endometrial biopsy under direct visualisation is associated with a significantly higher rate of sample adequacy and is comparable to blind endometrial sampling for the diagnosis of endometrial cancer and precancer. A large, well-designed, randomised controlled trial, is needed to confirm our findings.
